# Robotic-Assisted Laparoscopic Partial Nephrectomy for Multiple Synchronous Renal Tumors in a Solitary Kidney: A Case Report

**DOI:** 10.7759/cureus.94560

**Published:** 2025-10-14

**Authors:** Toni Franz, Anja Dietel, Doreen Trebst, Lars-Christian Horn, Jens-Uwe Stolzenburg

**Affiliations:** 1 Department of Urology, University of Leipzig, Leipzig, DEU; 2 Institute of Pathology, University of Leipzig, Leipzig, DEU

**Keywords:** 3d reconstruction, multiple kidney tumors, partial nephrectomy, robotic surgery, solitary kidney

## Abstract

We report the case of a 65-year-old female with multiple synchronous clear cell renal cell carcinomas (ccRCC) in a solitary kidney following prior radical nephrectomy of the contralateral side, presenting with five tumors ranging from 0.8 cm to 3.6 cm in size, distributed across all segments of the kidney as well as on both the anterior and posterior surfaces. Due to the complex tumor distribution, a patient-specific 3D model was created for preoperative planning to precisely assess the spatial relationship of the tumors to the main renal vessels, intrarenal vasculature, collecting system, and ureter. Given the pre-existing chronic kidney disease, nephron-sparing surgery was performed using a robotic-assisted laparoscopic approach, with intraoperative ultrasound and selective vascular clamping additionally employed to further optimize the resection strategy. All tumors were completely resected with negative margins. The postoperative course was uneventful; renal function remained stable, and dialysis was not required. The patient has remained cancer-free during follow-up. This case highlights the technical feasibility and clinical value of nephron-sparing surgery even in multifocal, anatomically complex renal tumors. The integration of advanced imaging modalities and individualized 3D surgical planning can improve oncological outcomes while preserving renal function, emphasizing the importance of a personalized, technology-assisted approach in contemporary renal tumor surgery.

## Introduction

Renal cell carcinoma (RCC) is among the most frequently diagnosed urological malignancies, ranking within the top 10 cancers in men and the top 15 in women worldwide [1]. Partial nephrectomy (PN) is considered the standard of care for T1a tumors and is strongly recommended by current European Association of Urology (EAU) guidelines for T1b lesions as well [2]. Nephron-sparing surgery has gained increasing importance, as it ensures both oncologic control and preservation of renal function. This aspect is particularly critical in patients presenting with multiple tumors in a single kidney, where complete tumor excision must be balanced against maximal preservation of functional parenchyma. Standard treatment with radical nephrectomy results in immediate end-stage renal disease and the need for lifelong dialysis. Nephron-sparing surgery (NSS), when technically feasible, is therefore the preferred approach to preserve renal function and quality of life, despite increased operative complexity [3,4].

The advent of robot-assisted surgery has substantially reduced the technical challenges associated with complex PN procedures [5,6]. Surgical success is commonly defined by the "trifecta" criteria, which include negative surgical margins, avoidance of major complications, and maintenance of renal function [7]. However, several tumor- and patient-related factors, such as proximity to the renal hilum or major vessels, endophytic growth, and adherent perinephric fat, can significantly compromise the achievement of these outcomes [8]. These difficulties are further amplified in the presence of multifocal disease, where surgical strategy must carefully balance oncologic radicality with nephron preservation.

Innovative technological approaches have been introduced to improve surgical planning and execution under these demanding conditions. Among them, virtual interactive three-dimensional (3D) modeling has emerged as a particularly promising tool.

## Case presentation

**Figure 1:**
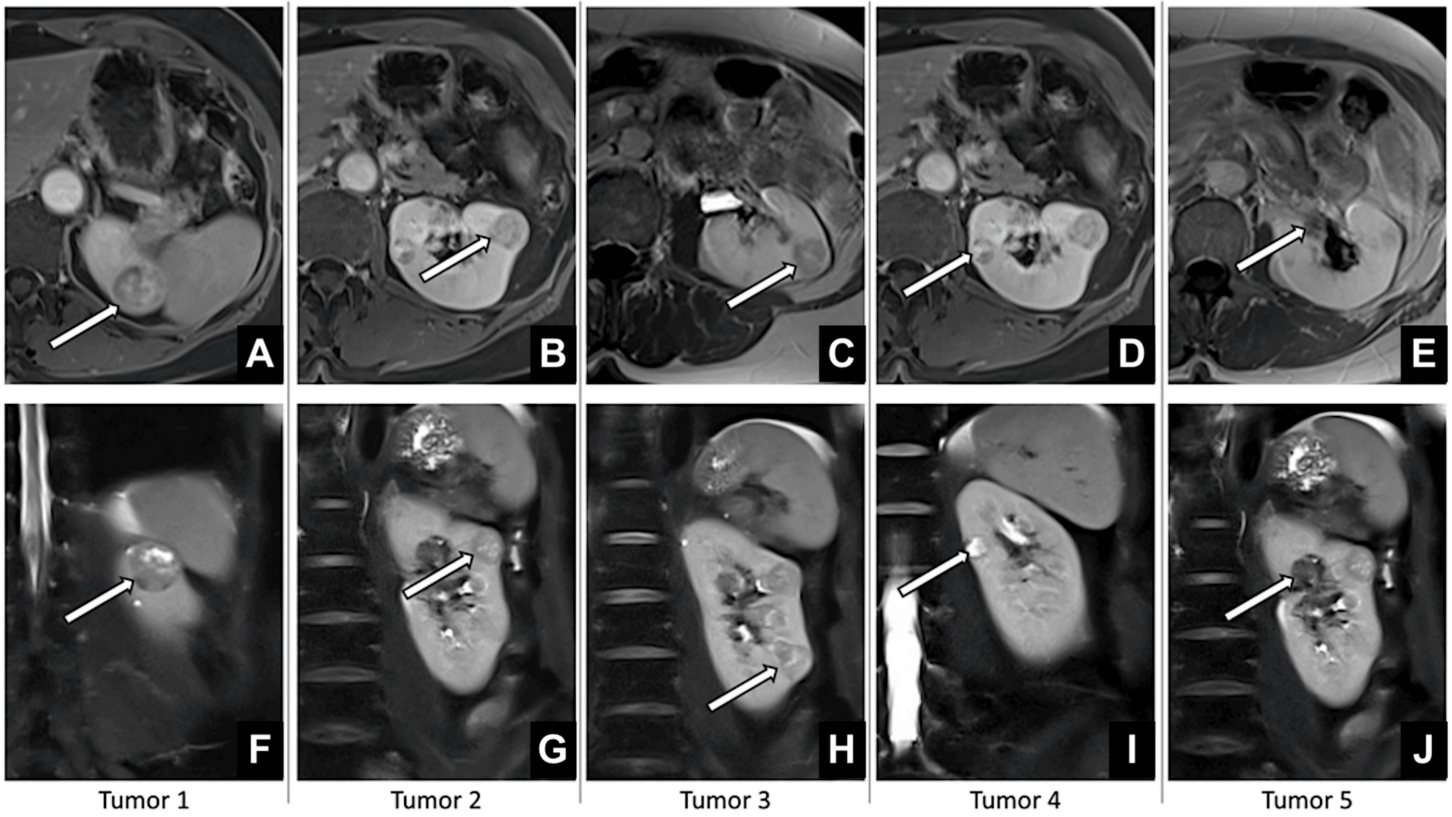
MRI of the abdomen demonstrating five renal tumors: (A-E) transverse view; (F-J) coronal view, each tumor indicated by a white arrow Image Credit: Toni Franz

A 65-year-old female with a history of right-sided radical nephrectomy for RCC three years prior was referred after routine follow-up imaging revealed multiple lesions in her solitary left kidney. Relevant comorbidities included arterial hypertension and rheumatoid arthritis. Laboratory investigations confirmed pre-existing chronic kidney disease, with a glomerular filtration rate (GFR) reduced to 52 mL/min and serum creatinine of 99 µmol/L. Contrast-enhanced magnetic resonance imaging demonstrated five enhancing renal masses, ranging from 8 to 36 mm in maximum diameter, distributed across all renal segments (Figure 1).

To the best of our knowledge, this represents the first reported case of PN involving at least five tumors in a solitary kidney. Given the patient's prior history of right-sided tumor nephrectomy, the possibility of a hereditary renal cancer syndrome should be considered.

Given the functional status of the solitary kidney, completion nephrectomy would have resulted in immediate dialysis dependence. Following an interdisciplinary discussion, the advantages and disadvantages of radical nephrectomy, PN, and interventional radiological procedures were discussed with the patient in a participatory decision-making process, with particular emphasis on the oncological perspective and quality of life. Finally, a nephron-sparing approach was planned.

**Figure 2:**
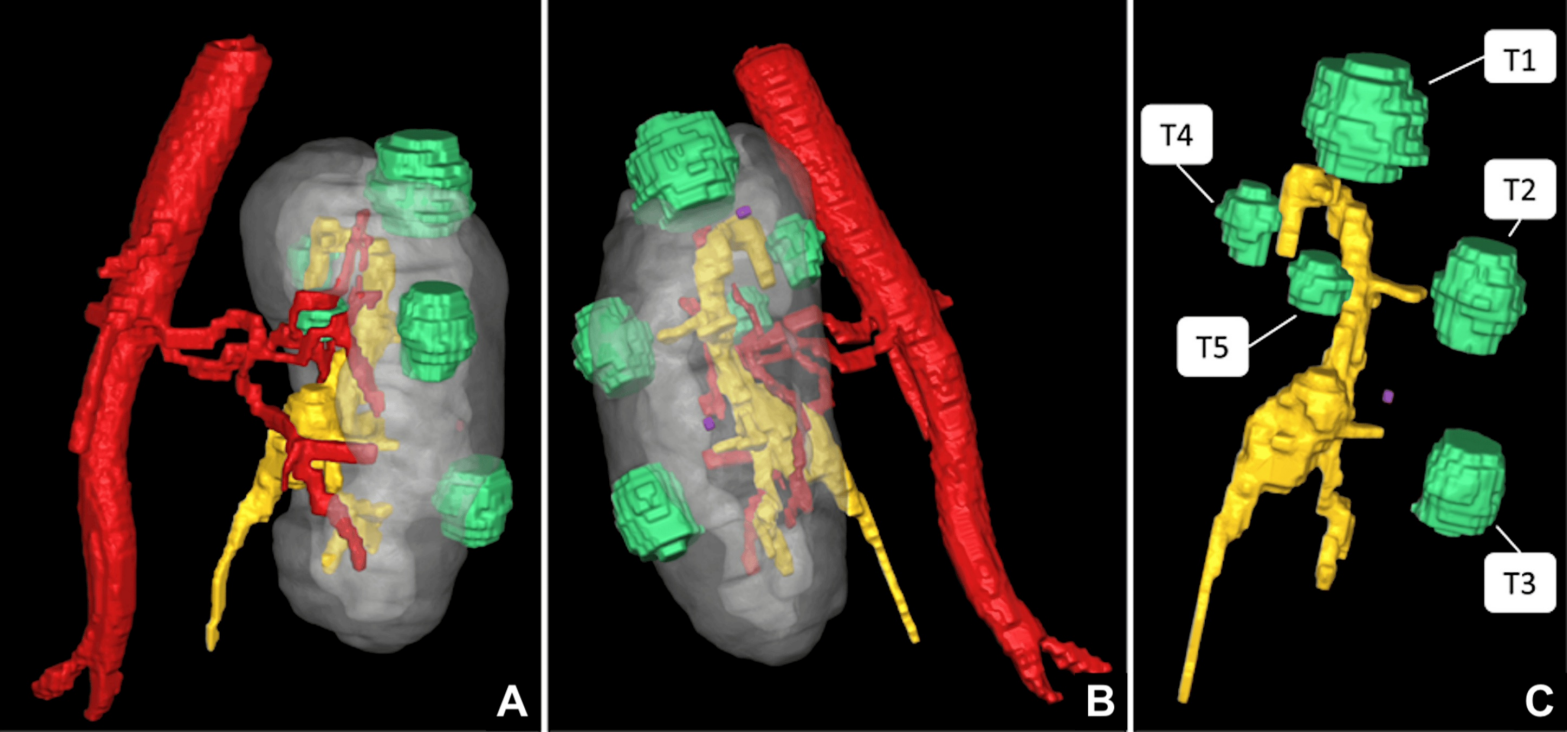
3D model of the solitary left kidney in (A) ventral, (B) lateral, and (C) parenchyma hidden views Image Credit: Toni Franz

The patient underwent robotic-assisted laparoscopic partial nephrectomy with preoperative three-dimensional (3D) modeling and augmented reality support. These 3D models were generated by Innersight Labs (Innersight Labs Ltd., London, UK) using Digital Imaging and Communications in Medicine (DICOM) datasets derived from computed tomography (CT) imaging for medical image segmentation (Figure 2). High-fidelity reconstructions were obtained as the CT slice thickness did not exceed 1.5 mm. The models could be accessed via a browser-based platform, allowing interactive visualization on a tablet both prior to and during surgery. In this complex case, we utilized the patient-specific 3D model to assist with preoperative planning, providing enhanced visualization of the adjacent vascular and calyceal anatomy. This enabled a more accurate assessment of the size, location, and depth of each renal lesion. Consequently, the 3D reconstruction helped us to better understand the patient's preoperative vascular architecture and to develop a more precise surgical strategy for robot-assisted partial nephrectomy (RAPN).

**Figure 3:**
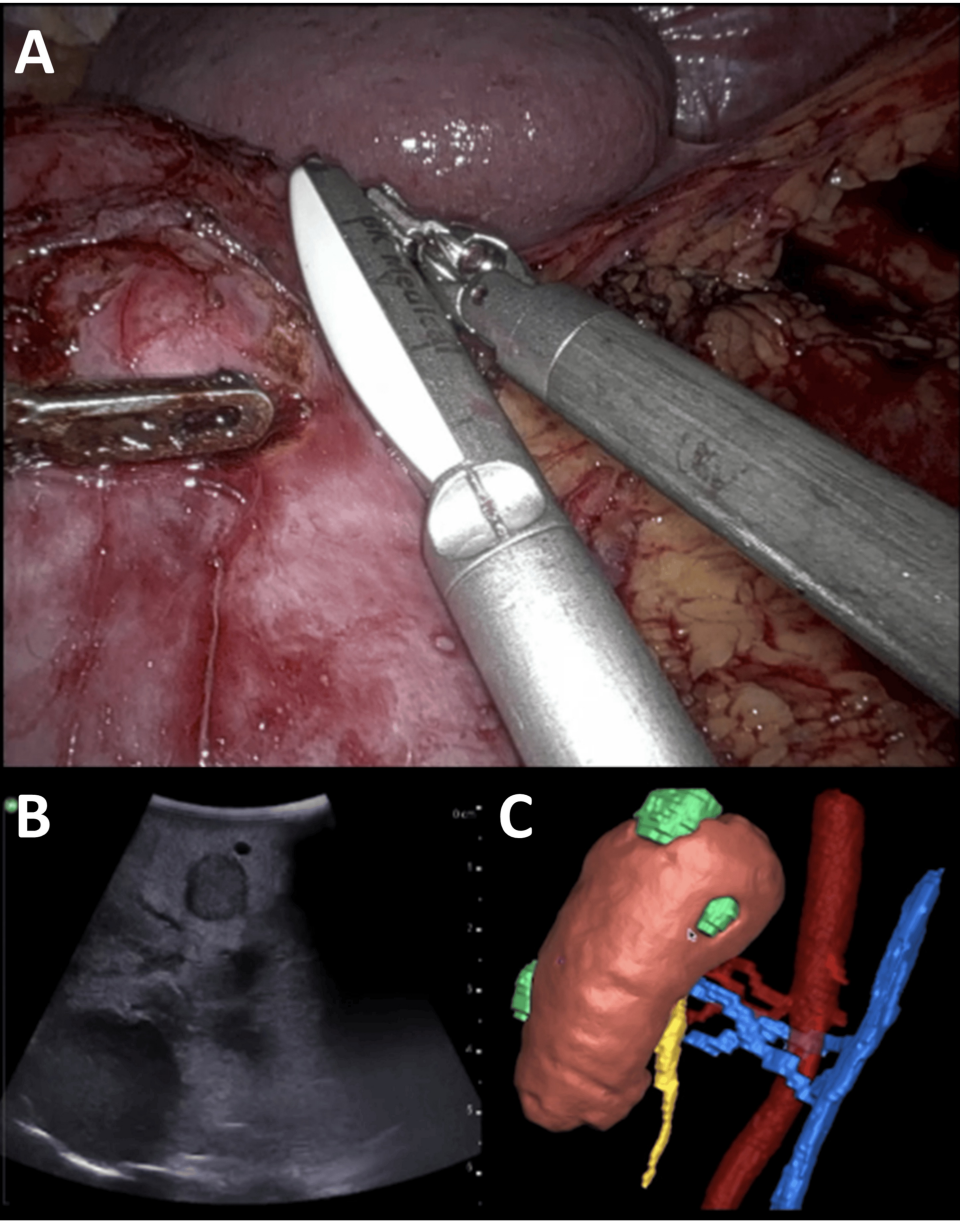
Intraoperative situs with real-time ultrasound guidance combined with the three-dimensional model. (A) Intraoperative view during robot-assisted partial nephrectomy. The needle driver is guiding the laparoscopic ultrasound probe directly onto the renal surface. (B) Laparoscopic ultrasound image depicting two of the five renal tumors. (C) Patient-specific three-dimensional kidney model reconstructed from preoperative CT imaging Image Credit: Toni Franz

**Figure 4:**
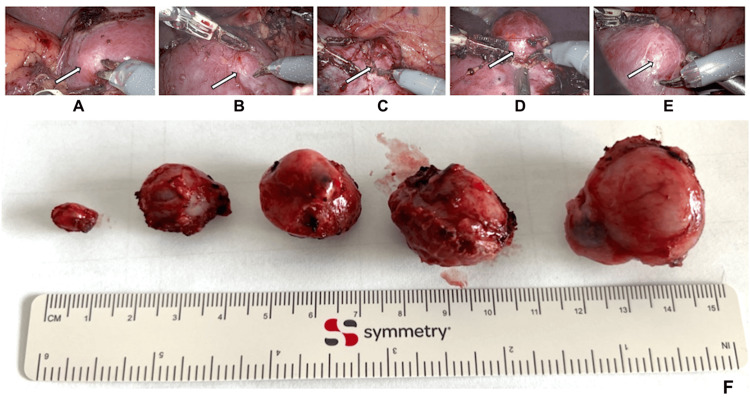
Intraoperative view showing the five tumors: (A) tumor 5, (B) tumor 4, (C) tumor 3, (D) tumor 2, (E) tumor 1, and (F) the surgical specimens Image Credit: Toni Franz

Intraoperative real-time ultrasound combined with the 3D model facilitated precise localization of all five tumors, which were enucleoresected (Figures 3, 4). Intraoperative real-time ultrasound combined with the 3D model facilitated precise localization of all five tumors, which were subsequently enucleoresected (Figures 3, 4). For this purpose, the laparoscopic ultrasound probe was carefully moved over the renal surface to identify each individual lesion and to assess its spatial relationship to adjacent critical structures such as segmental vessels, the collecting system, and the renal hilum. In parallel, the patient-specific 3D model was interactively manipulated to achieve congruence between the virtual reconstruction and the intraoperative anatomy, providing continuous spatial orientation throughout the procedure. Once all lesions had been identified, the tumor margins were delineated using live ultrasound imaging projected onto the Da Vinci robotic console and were gently marked on the renal surface with low-intensity monopolar cautery to guide precise resection. Subsequently, three smaller lesions were excised clampless without ischemia, whereas the two larger masses were resected under a cumulative warm ischemia time of 10:30 minutes. Parenchymal defects were reconstructed using suturing with the sliding clip technique. Total operative time was 160 minutes, with an estimated blood loss of 220 mL.

Postoperatively, renal function initially declined from a preoperative GFR of 52 mL/min/1.73 m² (creatinine 99 µmol/L) to 16 mL/min/1.73 m² (creatinine 276 µmol/L), but subsequently recovered to a stable level with a GFR of 37 mL/min/1.73 m² (creatinine 138 µmol/L). The recovery of the patient was uneventful. The postoperative ultrasound did not show any abnormality. The surgical drain was removed on the second postoperative day. Histopathological examination revealed moderately differentiated clear cell renal cell carcinomas, measuring 0.8 cm, 2.1 cm, 2.2 cm, 2.0 cm, and 3.6 cm in maximum diameter (Figure 4). According to the TNM classification (2017), the tumor was staged as pT1a(m) pNX M0, L0 V0 Pn0, corresponding to UICC stage I, grade 2, with negative resection margins (R0). Genetic evaluation revealed no pathological findings of direct clinical relevance. One and a half years after the surgery, the patient is still cancer-free with a stable renal function (eGFR 35 mL/min, serum creatinine 147 µmol/L).

## Discussion

Multifocal RCC in a solitary kidney presents significant therapeutic dilemmas. The primary goal is to achieve complete oncological control while preserving maximal renal function. Robotic-assisted laparoscopic partial nephrectomy (RAPN) offers precise tumor excision with reduced warm ischemia time, even in multifocal cases [9,10]. In our patient, the procedure successfully avoided dialysis initiation and achieved oncologically safe outcomes.

Previous reports have shown that RAPN in solitary kidneys can achieve excellent functional preservation with low complication rates, even for complex or multiple lesions [11,12]. In a study by Dawidek et al., 73 high-risk patients with a solitary kidney demonstrated a five-year end-stage renal disease-free survival rate of 89%. The oncological outcomes in the high-risk cohort showed five-year local recurrence-free, cancer-specific, and overall survival rates of 83%, 80%, and 77%, respectively [13]. The working group led by Soputro et al. investigated the outcomes in 86 patients who underwent PN in the setting of a solitary kidney. The solitary kidney cohort demonstrated similarly favorable long-term functional outcomes compared to patients with bilaterally functioning kidneys. Notably, and unsurprisingly, the solitary kidney group exhibited a higher risk of postoperative acute kidney injury (AKI) (solitary vs. non-solitary: p < 0.001) [14]. Grosso et al. also support the conclusion that RAPN represents an effective treatment option for renal tumors in solitary kidneys, providing a balance between oncological control and preservation of renal function. According to their findings, global ischemic clamping (full clamping) and the patient's overall physical condition are relevant factors influencing outcomes, underscoring the importance of careful patient selection and individualized surgical strategies [15].

Early detection through follow-up imaging and timely surgical intervention are crucial to enable a nephron-sparing approach in such challenging scenarios. Three-dimensional reconstructions and augmented reality (AR) can improve spatial orientation in complex renal surgery and may help to reduce ischemia time; however, their clinical value still requires further scientific validation [16,17]. The use of 3D models has already been shown to enhance both preoperative and intraoperative precision in planning RAPN. By enabling an individualized, patient-specific approach, 3D visualization allows surgeons to move beyond standardized "one-size-fits-all" strategies, optimize tumor resection, and identify the most appropriate arterial clamping sites while visualizing perfusion territories and improving vascular control [18,19]. Moreover, AR-assisted laparoscopic videos that integrate updated preoperative imaging with the Da Vinci robotic platform provide additional benefits by enhancing the accuracy of tissue resection, minimizing the removal of adjacent healthy tissue, and maintaining adequate surgical margins [20].

## Conclusions

This case demonstrates the technical feasibility and clinical benefit of RAPN in the challenging scenario of multiple synchronous renal tumors in a solitary kidney. Despite the complexity of resecting five distinct lesions, NSS was successfully performed with acceptable ischemia time, preservation of renal function, and oncologically safe margins. The postoperative course and functional recovery underscore that, with careful patient selection, advanced intraoperative imaging, and robotic assistance, nephron-sparing approaches can be achieved even in highly complex multifocal cases. These findings support the notion that organ-preserving strategies should be prioritized whenever technically feasible, as they offer a meaningful chance of avoiding dialysis dependence while maintaining oncological efficacy.
